# Repetitive Negative Thinking and Interpretation Bias in Pregnancy

**DOI:** 10.32872/cpe.v2i4.3615

**Published:** 2020-12-23

**Authors:** Colette R. Hirsch, Frances Meeten, Calum Gordon, Jill M. Newby, Debra Bick, Michelle L. Moulds

**Affiliations:** a Institute of Psychiatry, Psychology and Neuroscience, King’s College London, London, United Kingdom; b School of Psychology, University of Sussex, Sussex, United Kingdom; c School of Psychology, University of New South Wales, Sydney, Australia; d Black Dog Institute, Hospital Road Randwick, New South Wales, Sydney, Australia; e Warwick Clinical Trials Unit, University of Warwick, Coventry, United Kingdom; Philipps-University of Marburg, Marburg, Germany

**Keywords:** perinatal mental health, repetitive thinking, worry, interpretation bias, pregnancy

## Abstract

**Background:**

Repetitive negative thinking (RNT; e.g., worry about the future, rumination about the past) and the tendency to interpret ambiguous information in negative ways (interpretation bias) are cognitive processes that play a maintaining role in anxiety and depression, and recent evidence has demonstrated that interpretation bias maintains RNT. In the context of perinatal mental health, RNT has received minimal research attention (despite the fact that it predicts later anxiety and depression), and interpretation bias remains unstudied (despite evidence that it maintains depression and anxiety which are common in this period).

**Method:**

We investigated the relationship between RNT, interpretation bias and psychopathology (depression, anxiety) in a pregnant sample (n = 133). We also recruited an age-matched sample of non-pregnant women (n = 104), to examine whether interpretation bias associated with RNT emerges for ambiguous stimuli regardless of its current personal relevance (i.e., pregnancy or non-pregnancy-related).

**Results:**

As predicted, for pregnant women, negative interpretation bias, RNT, depression and anxiety were all positively associated. Interpretation bias was evident to the same degree for material that was salient (pregnancy-related) and non-salient (general), and pregnant and non-pregnant women did not differ. RNT was associated with interpretation bias for all stimuli and across the full sample.

**Conclusion:**

Our findings highlight the need to further investigate the impact of interpretation bias in pregnant women, and test the effectiveness of interventions which promote positive interpretations in reducing RNT in the perinatal period.

## Abstract (highlights)

- A tendency to make negative interpretations was investigated in pregnant women for the first time.
- Negative interpretation bias was associated with repetitive negative thinking.
- Interpretation bias extended to pregnancy related information for pregnant and non-pregnant women.
- Reducing negative interpretation bias in pregnant women could be useful.

Repetitive negative thinking (RNT) plays a role in the onset and maintenance of depression (Nolen-Hoeksema et al., 2008), and is transdiagnostic such that it is evident in a range of disorders, including anxiety (Ehring & Watkins, 2008). RNT refers to thinking that is negative, perseverative and difficult to control, whether about the past (rumination) or future (worry) (Samtani & Moulds, 2017). Perinatal depression and anxiety are common. One in four pregnant women report mental health problems (Howard et al., 2018), the most common being anxiety and depression, and they commonly persist into early motherhood. Given the role of RNT in predicting and maintaining both anxiety and depression, it is surprising that RNT in the perinatal period has only recently received research attention (e.g., DeJong et al., 2016; Moulds et al., 2018; Newby et al., 2019).

Consistent with the broader RNT literature, there is growing evidence that antenatal RNT predicts perinatal mental health problems. Schmidt et al. (2016) reported that RNT in the first trimester predicted depression and anxiety in the third trimester (Schmidt et al., 2016), and that RNT interacts with other factors (e.g., level of social functioning; O’Mahen et al., 2010; perfectionism; Egan et al., 2017) to predict postnatal depression. In another longitudinal study, RNT in late pregnancy (i.e., third trimester) predicted change in depression symptoms from the third trimester to 8 weeks postpartum, an association that was not moderated by initial levels of depression (Barnum et al., 2013). Building on correlational findings, there is experimental evidence that RNT maintains postnatal difficulties. In a sample of new mothers, RNT impaired problem-solving ability and reduced confidence in problem-solving capacity (O’Mahen et al., 2015). Similarly, in women with postpartum GAD, RNT reduced responsivity to infants – suggesting a key role for RNT in mother-infant bonding (Stein et al., 2012). Taken together, these findings highlight that RNT plays a key detrimental role in the perinatal context.

Interpretation bias – the tendency to draw negative conclusions from ambiguous information - is a transdiagnostic cognitive process evident across emotional disorders (Hirsch et al., 2016). Interpretation bias often focuses on an individual’s core clinical concern. For example, individuals with panic disorder (Stopa & Clark, 2000) and social anxiety disorder (Amin et al., 1998) demonstrated a more negative interpretation bias for ambiguously threatening information which was central to their clinical problem (i.e., panic and socially-related material, respectively) relative to both individuals with other forms of anxiety, and non-clinical control participants. This content specificity is also evident in children who experience higher levels of anxiety specific to particular fears (e.g. social anxiety, separation anxiety, fear of spiders; Mobach et al., 2019). Relatedly, Everaert et al. (2017) hypothesized that the personal relevance of material may be key to observing interpretation bias, such that the material has to relevant the person themselves in order to be processed in a biased manner.

Interpretation bias is associated with different forms of RNT (Krahé et al., 2019) across the population, and individuals with GAD and depression demonstrate particularly high levels of this bias. There is evidence that targeting (i.e., reducing) a negative interpretation bias has the downstream effect of reducing RNT. For example, training individuals with GAD to interpret ambiguous information as benign (rather than negative) reduced worry frequency (Hayes et al., 2010). In addition, there is evidence that training in generating positive interpretations leads to reduced RNT and anxiety in individuals with high levels of RNT (Hirsch, Krahé, Whyte, Bridge, et al., 2020), as well as those with clinical anxiety and/or depression (Hirsch et al., 2018; Hirsch, Krahé, Whyte, Krzyzanowski, et al., 2020). Moreover, improvements in worry, rumination, anxiety and depression are mediated by decreases in interpretation bias, consistent with it being the mechanism of change (Hirsch, Krahé, Whyte, Krzyzanowski, et al., 2020).

To date, no research has investigated interpretation bias in the perinatal context. It is therefore unknown whether women in the perinatal period have a tendency to draw negative conclusions when presented with ambiguity - and if such a bias does exist - whether it is associated with levels of depression and anxiety, as well as RNT. Further, if such a bias is indeed present, it will be both theoretically and clinically informative to establish whether this mechanism also applies to pregnancy-related ambiguous stimuli (e.g., the outcome of a foetal scan) which would be particularly salient and personally relevant for pregnant but not non-pregnant women. This speaks to a wider conceptual question regarding the nature of interpretation bias underlying RNT: is it a general mechanism that applies to any ambiguity, whether or not it is personally relevant and salient? In order to answer this question, we recruited a sample of matched non-pregnant women and examined whether this general bias also operates for ambiguous material that is not likely to be personally relevant (i.e., is pregnancy-related). That is, if interpretation bias that is associated with RNT is reduced by lack of current personal relevance, pregnancy-related material would elicit a weaker bias in non-pregnant women with high levels of RNT compared to pregnant women with high levels of RNT. Alternatively, if such an interpretation bias operates on ambiguously threatening material irrespective of current personal relevance, we would predict an association between RNT and this bias regardless of pregnancy status or personal relevance of the material (i.e., pregnancy-related versus general ambiguity).

In sum, extant findings confirm that interpretation bias and RNT are interrelated, and there is emerging evidence that RNT is a key cognitive process in the context of perinatal mental health. However, it remains unknown whether negative interpretation bias is associated with depression and anxiety in the perinatal period. Furthermore, the possibility that RNT is correlated with interpretation bias in this period has not been examined to date. Accordingly, our first goal was to investigate associations between interpretation bias, RNT (as a trait tendency, as well as specific types of RNT including depressive rumination and worry), as well as symptoms of psychopathology (anxiety, depression) in a community sample of pregnant women. We hypothesised significant positive relationships between RNT, interpretation bias, depression and anxiety symptoms.

Second, we were interested in whether interpretation bias associated with RNT emerges for ambiguous stimuli regardless of its current personal relevance. We recruited a sample of age-matched women who were not pregnant, and thus for whom pregnancy-related materials were not likely to be personally relevant. We then examined the association between levels of RNT and interpretation bias for pregnancy-related and general (non-pregnancy-related) materials in samples of both pregnant and non-pregnant women. This enabled us to establish whether interpretation bias underlying RNT operates on all ambiguously threatening material, irrespective of personal relevance.

## Method

### Participants

We recruited 140 pregnant and 107 non-pregnant female participants who were 25-40 years of age, fluent in English and based in the UK. Pregnant participants were eligible to take part if they were at least 16 weeks gestation, and had not previously experienced a stillbirth. Non-pregnant participants were eligible if they were not currently trying to fall pregnant, and had not experienced a stillbirth in the past. Participants were recruited through social media, online message boards, and the King’s College London research circular. The final sample was comprised of 133 pregnant and 104 non-pregnant women^1^1Nine participants were removed from analysis with a score on the Recognition Test comprehension questions 2.5 standard deviations below the group mean. An additional participant was removed from analysis for having no grammatically correct sentences in the Scrambled Sentences Test. Seven pregnant and three non-pregnant participants were removed from analysis.. See Table 1 for participant demographics.

**Table 1:** Demographic Characteristics

Baseline Characteristic	Pregnant sample (*N* = 133)		Non-pregnant sample (*N* = 104)		Statistical test and significance value
	*M*	*SD*	*M*	*SD*	*t* (235) = 5.04, *p* < .001
**Age**	32.64	3.68	30.12	4.0	
Nationality %	*n*	%	*n*	%	χ^2^ (2) = 15.94, *p* < .001
British	123	92.5	77	74.0	
Other European	4	3.0	16	15.4	
World	6	4.5	11	10.6	
Highest level of education	*n*	%	*n*	%	χ^2^ (4) = 4.61, *p* = .33
Secondary	26	19.5	13	12.5	
Bachelor	52	39.1	37	35.6	
Master	33	24.8	37	35.6	
Doctoral	7	5.3	7	6.7	
Other	15	11.3	10	9.6	
Marital status	*n*	%	*n*	%	
Single, never married	2	1.5	24	23.1	χ^2^ (3) = 56.63, *p* < .001
In a relationship	30	22.6	46	44.2	
Married /domestic partnership	100	75.2	31	29.8	
Separated, divorced, widowed	1	0.8	3	2.9	
Number of children	*n*	%	*n*	%	χ^2^ (3) = 24.63, *p* < .001
None	46	34.6	66	63.5	
One	65	48.9	19	18.3	
Two	16	12.0	14	13.5	
Three or more	6	4.5	5	4.8	
**English as a native language**	123	92.5	84	80.8	χ^2^ (1) = 7.24, *p* = .007

### Materials and Measures

#### Demographic Questions

Participants completed a number of demographic questions regarding age, nationality, level of education, relationship status, number of children and English fluency. Participants were also asked whether they were currently pregnant, and if they responded yes, asked to indicate number of weeks gestation, and whether they had previously experienced a stillbirth.

#### Interpretation Measures

##### Scrambled Sentences Test (SST)

This task was employed by Hirsch et al. (2018) and Hirsch, Krahé, Whyte, Bridge, et al. (2020), adapted from Wenzlaff and Bates (1998, 2000). Participants unscramble six words presented in a random order into a grammatically correct sentence of either positive or negative valence. Participants were given 20 sentences, equally divided between worry themes and depressive rumination themes, and asked to unscramble as many as possible in five minutes whilst holding a six-digit number in mind (which increased cognitive load; see Wenzlaff & Bates, 1998, 2000)^2^2See Appendix A in the Supplementary Materials for sample items.. An index of interpretation bias was created by dividing the number of grammatically correct positively unscrambled sentences by the total number of grammatically correct unscrambled sentences. Index scores range from 0 to 1, higher scores denote a more positive interpretation bias. The SST had good internal consistency α = .86, which is comparable to that reported in a recent validation paper where two SST lists of worry and depression items were examined with α = .77 and α = .92 respectively (Krahé et al. 2020).

##### Recognition Test (RT)

This test was based on that used by Mathews and Mackintosh (2000). Materials included items related to two themes – pregnancy related and general (non-pregnancy) related. General materials were drawn from worry and rumination recognition test materials used by Hirsch, Krahé, Whyte, & Bridge, et al. (2020), while the pregnancy materials were developed for the current study from interviews with four pregnant women^3^3See Appendix B in the Supplementary Materials for sample items.. The RT has two phases: in the first, participants read 21 ambiguous scenarios and answered a comprehension question after each scenario. In the second section, after all scenarios had been read, participants were presented with the title of each scenario, followed by four statements presented in a random order. Participants rated how similar each statement was to the scenario they read on a 4-item Likert scale from 1 (*very different in meaning*) to 4 (*very similar in meaning*). Two of these statements resolved the previously read ambiguous scenario in either a positive or negative way, consistent with the story (targets). The remaining two statements were positively and negatively valenced, but were not realistic interpretations of the story (foils; included as filler items).

Twenty-one scenarios were equally split between worry and rumination themes, and themes relating to pregnancy. Worry and rumination items were a subset of those used by Hirsch, Krahé, Whyte, Bridge, et al. (2020). An interpretation bias index was created for each participant by subtracting mean ratings for negative targets from mean ratings for positive targets, with a higher score denoting a more positive interpretation bias. Pregnancy interpretation bias index (7 items), general interpretation bias index (14 items) and total interpretation bias index (including both pregnancy and general items) were computed. Split half reliability was high, Spearman-Brown coefficient for Negative Targets and Positive Targets respectively was .83 and .85.

#### Questionnaire Measures

##### Repetitive Thinking Questionnaire (RTQ-T [trait])

The 10-item RTQ-T (trait) (McEvoy, Thibodeau, & Asmundson, 2014) measures trait repetitive negative thinking. Participants rate the extent to which each item (e.g., *‘I have thoughts or images about all my shortcomings, failings, faults, mistakes’*) is true for them when they are distressed or upset. The RTQ possesses good internal consistency, convergent and divergent validity (Mahoney, McEvoy, & Moulds, 2012). Present sample Cronbach’s α = .92.

##### Penn State Worry Questionnaire (PSWQ)

The 16-item PSWQ (Meyer, Miller, Metzger, & Borkovec, 1990) measures worry (example item: *‘My worries overwhelm me’*). Participants rate the extent to which each item is typical of their experience. The PSWQ has good test-retest reliability (Meyer et al., 1990) and good convergent and discriminant validity (Brown, Antony, & Barlow, 1992). Present sample Cronbach’s α = .83.

##### Ruminative Response Scale (RRS)

Depressive rumination was assessed using the 22-item measure RRS (Nolen-Hoeksema & Morrow, 1991). Participants rate the extent to which they engage in a range of responses when they feel sad, down or depressed (e.g., ‘*think about how alone you feel*’). The RRS has good internal consistency (Treynor, Gonzalez, & Nolen-Hoeksema, 2003) and test-retest reliability (Just & Alloy, 1997). Present sample Cronbach’s α = .94.

##### Generalized Anxiety Disorder 7-item scale (GAD-7)

The 7-item GAD-7 (Spitzer et al., 2006) questionnaire measures anxiety symptoms over the past 2 weeks (example item: ‘*Feeling nervous, anxious, or on edge?*). The GAD-7 is a reliable and valid measure of anxiety in the general population (Löwe et al., 2008). Present sample Cronbach’s α = .92.

##### Patient Health Questionnaire 9

The 9-item PHQ-9 (Kroenke & Spitzer, 2002) measures depression symptoms in the previous 2 weeks. The PHQ-9 is a reliable and valid measure of depression severity (Kroenke, Spitzer, & Williams, 2001). Present sample Cronbach’s α = .88.

##### Perinatal Anxiety Screening Scale

Pregnant participants completed the 31-item PASS (Somerville et al., 2014), which measures anxiety in antenatal and postpartum women. Participants indicate how often they experience each item (e.g., ‘*Fear that harm will come to the baby*’) in the past month. The PASS has good reliability and validity (Somerville et al., 2014). Present sample Cronbach’s α = .95.

##### Edinburgh Postnatal Depression Scale (EPDS)

The 10-item EPDS (Cox, Holden, & Sagovsky, 1987) was used to assess depression symptoms in pregnant participants. It possesses a high level of test-retest reliability (Kernot, Olds, Lewis, & Maher, 2015), and good validity (Gibson, McKenzie-McHarg, Shakespeare, Price, & Gray, 2009). Present sample Cronbach’s α = .89.

### Procedure

The survey was hosted on the Qualtrics platform. Participants were asked to complete the survey in one sitting, at a time they could be free from distractions. Both groups of participants completed the same core survey (questionnaires, SST, RT), pregnant participants completed two additional pregnancy-specific questionnaires (PASS, EPDS). The survey took 35-40 minutes to complete. Upon completion participants received a £5 Amazon voucher. The study was approved by the King’s College London Research Ethics Committee (approval number: HR-17/18-5735). Participants provided consent electronically.

## Results

Mean questionnaire scores by group are presented in Table 2.

**Table 2:** Descriptive Statistics for Questionnaires and Bias Measures by Group

Measures	Pregnant group (*N* = 133)		Non-pregnant group (*N* = 104)		*t*-test and significance value
	*M*	*SD*	*M*	*SD*	
Questionnaire					
RTQ	29.78	9.53	30.14	9.44	*t* (235) = 0.29, *p = .*77
GAD7	7.04	5.32	6.84	6.22	*t* (202.894)^a^ = 0.26, *p = .*79
PHQ9	8.09	5.66	7.56	6.41	*t* (206.795)^a^ = 0.67, *p = .*51
PSWQ	52.16	14.02	53.57	14.90	*t* (235) = 0.75, *p = .*46
RRS	45.61	13.24	51.53	14.60	*t* (235) = 3.27, *p = .*001
PASS	30.23	18.25	–		–
EPDS	9.26	5.77	–		–
Interpretation bias measures					
RT pregnancy items	0.42	0.78	0.36	0.77	*t* (235) = 0.57, *p =* .57
RT general items	0.68	0.66	0.60	.73	*t* (235) = 0.85, *p =* .39
RT all items	0.59	0.65	0.52	0.68	*t* (235) = 0.82, *p =* .42
SST	0.72	0.20	0.69	0.23	*t* (235) = 1.17, *p =* .24

*Note.* PSWQ = Penn State Worry Questionnaire; RRS = Ruminative Response Scale; RTQ = Repetitive Thinking Questionnaire; GAD7 = Generalised Anxiety Disorder Questionnaire; PHQ9 = Patient Health Questionnaire; PASS = Perinatal Anxiety Screening Scale; EPDS = Edinburgh Postnatal Depression Scale; RT = Recognition Test; SST = Scrambled Sentences Test.

^a^Equal variances not assumed.

Mean scores on questionnaire measures (RTQ, GAD7, PHQ9, PSWQ) did not differ between groups (*p*s > .05), except on the RRS, where the non-pregnant group reported significantly higher levels of rumination, *t*(235) = 3.27, *p = .*001, *r* = .21.

### Is There an Association Between Interpretation Bias and Repetitive Negative Thinking, and Anxiety and Depression in a Sample of Pregnant Women?

To examine whether levels of RNT, worry and rumination were associated with a more negative interpretation bias in pregnant women, we examined correlations between the RNT measures and the behavioural measures of interpretation bias (SST, RT pregnancy items, RT general items, and all RT items collapsed)^4^4In the pregnant sample, the two interpretation bias measures, the RT (all items) and the SST were significantly correlated (*r* = .33, *p* < .001). (see Table 3 for correlations by group). Trait repetitive thinking (measured by the RTQ) was significantly negatively correlated with SST index (*r* = -.61, *p* < .001). Anxiety (measured by the GAD7; *r* = -.63, *p* < .001), worry (measured by the PSWQ; *r* = -.67, *p* < .001), depression (measured by the PHQ9; *r* = -.62, *p* < .001), and depressive rumination (measured by the RRS; *r* = -.72, *p* < .001) were also significantly negatively correlated with the SST.

**Table 3:** Correlations Between RNT and Interpretation Bias Measures (RT, SST) in Pregnant and Non-Pregnant Participants

Questionnaires	RT index			SST index
	Pregnancy items	Worry items	All items	
Pregnant group				
RTQ	-.24**	-.25**	-.27**	-.61**
GAD7	-.14	-.24**	-.22*	-.63**
PHQ9	-.24**	-.29**	-.30**	-.62**
PSWQ	-.16	-.24**	-.23**	-.67**
RRS	-.09	-.21*	-.18*	-.72**
Non-pregnant group				
RTQ	-.18	-.22*	-.22*	-.56**
GAD7	-.12	-.12	-.13	-.61**
PHQ9	-.15	-.18	-.18	-.68**
PSWQ	-.23*	-.24*	-.26**	-.64**
RRS	-.25*	-.21*	-.24*	-.66**

*Note.* RTQ = Repetitive Thinking Questionnaire; GAD7 = Generalised Anxiety Disorder Questionnaire; PHQ9 = Patient Health Questionnaire; PSWQ = Penn State Worry Questionnaire; RRS = Ruminative Response Scale; RT = Recognition Test; SST = Scrambled Sentences Test. *p < .05. **p < .01.

For the Recognition Test (RT), trait repetitive thinking (RTQ) was significantly negatively correlated with RT index (all items) (*r* = -.27, *p* = .002). Anxiety (*r* = -.22, *p* = .01) and worry (*r* = -.23, *p* = .008), depression (*r* = -.30, *p =* .001), and depressive rumination (*r* = -.18, *p* = .04) were also significantly negatively correlated with RT. To investigate bias specificity, we calculated the RT index for general and pregnancy-related items separately and examined correlations between both of these indices and self-report measures. The RT index for general items was significantly negatively correlated with RNT (*r* = -.25, *p* = .003), anxiety (*r* = -.24, *p* = .005), worry (*r* = -.24, *p* = .005), depression (*r* = -.29, *p* = .001), and depressive rumination (*r* = .21, *p* = .02). For the RT index comprised of pregnancy items, there was a significant negative correlation between RNT (*r* = -.24, *p* = .006) and depression (*r* = -.24, *p =* .006). No other associations were significant.

### Does Interpretation Bias Associated With RNT Emerge for Ambiguous Stimuli Regardless of its Current Personal Relevance?

To examine whether interpretation bias associated with RNT emerges for ambiguous stimuli regardless of its current personal relevance, we examined interpretation bias for pregnancy-related and general stimuli in samples of pregnant and non-pregnant women. We conducted a 2 group (pregnant vs. non-pregnant) x 2 RT material type (pregnancy-related vs. general) mixed model ANCOVA with repeated measures on the second factor and interpretation bias as the dependent variable. To examine whether trait RNT was associated with interpretative bias irrespective of group, RTQ-trait scores were included as a covariate. There was no significant main effect of group, *F*(1, 234) = 0.52, *p* = .47, $\eta_{p}^{2}$ = .002. There was no significant main effect of material type, *F*(1, 234) = 3.60, *p* = .06, $\eta_{p}^{2}$ = 0.02, however this effect approached significance, but with a small effect size. Examination of the means suggested that regardless of group (pregnant vs. non-pregnant), when RTQ was included in the model as a covariate, the RT positivity index was higher for general items (*M =* 0.64, *SE =* 0.04) than for pregnancy-related items (*M =* 0.40, *SE =* 0.05). There was no interaction of group and material type, *F*(1, 234) = 0.06, *p* = .81, $\eta_{p}^{2}$ < .001. Trait repetitive negative thinking (RTQ) was a significant covariate, indicating that trait RNT had a significant relationship with positivity index ratings (as measured by the RT) regardless of group or material type, *F*(1, 234) = 14.92, *p* < .001, $\eta_{p}^{2}$ = .06^5^5We re-ran the ANCOVA with RRS ratings included in the model as a covariate alongside RNT. The effects remain as described above and the RRS was not a significant covariate in the model (*p* = .36). However, we interpret this result with caution given significant group differences on RRS scores between the two groups at baseline (Field, 2009)..

## Discussion

We sought to establish whether there are associations between negative interpretation bias, RNT and symptoms of depression and anxiety in the perinatal period. Furthermore, if an interpretation bias is present, we sought to examine whether pregnant and non-pregnant women exhibit similar levels of interpretation bias for both general (likely to be personally salient for both groups) and pregnancy-related (likely to be only salient for pregnant but not pregnant women) ambiguous stimuli. Clarifying this would speak to the question of whether interpretation bias is lower for non-personally relevant information. In pregnant women, we found negative associations between two behavioural measures of interpretation bias, RNT, and psychopathology symptoms; that is, the more negative one’s interpretation bias, the higher their levels of RNT and symptoms of depression and anxiety. Regarding personal relevance, pregnant and non-pregnant women did not differ in their negative interpretation bias, irrespective of material type (pregnancy-related or general). Rather, trait RNT predicted interpretation bias regardless of pregnancy status or personal relevance of material focus.

It is noteworthy that mean scores on the self-report measures were relatively high in the current sample. Importantly, however, (with the exception of the RRS), the pregnant and non-pregnant groups were nonetheless matched. Thus, whilst our findings emerged in the context of high levels of psychopathology and RNT for a community sample, the fact that our groups were comparable nonetheless renders our between-group comparisons meaningful. That said, we acknowledge that the pregnant participants reported significantly lower levels of depressive rumination relative to their non-pregnant counterparts.

Our findings are theoretically informative, demonstrating that a bias to negatively interpret ambiguous stimuli also extends to women in the perinatal period, and that this bias is associated with psychological symptoms and RNT. Moreover, the bias is not influenced by personal relevance such that it was elicited by both pregnancy-related and general non-pregnancy-related material for women irrespective of pregnancy status. This suggests that the tendency to generate negative interpretations for those with higher levels of RNT may be applied to whatever ambiguity an individual encounters; the negative interpretation then has the potential to trigger further negative thoughts which may encompass other ambiguity and as such trigger new bouts of RNT which can then be perpetuated via further negative interpretations (Hirsch & Mathews, 2012; Hirsch et al., 2016). Furthermore, if these findings are replicated in those suffering from generalised anxiety disorder, it may help explain how these individuals end up worrying about so many new topics as soon as they encounter them, given that negative interpretations will trigger and maintain worry about a wide range of topics.

These results also have implications for the prevention of perinatal depression and/or anxiety, and suggest the potential clinical utility of offering interventions which effectively reduce cognitive biases, including cognitive behavioural therapy (CBT) and antidepressant medication. In addition, the findings suggest the potential utility of offering CBM-I targeting interpretation bias to vulnerable pregnant women (i.e., those with a history of psychopathology) in order to reduce RNT and associated psychological symptoms in the antenatal period. Given the generalised (i.e., rather than pregnancy-specific) nature of interpretation bias observed in our sample, such preventive interventions could utilise CBM-I materials employed in our previous work (e.g., Hirsch et al., 2018; Hirsch, Krahé, Whyte, Bridge, et al., 2020) to train pregnant women to generate positive interpretations, without the need for adaptation. However, if multi-session CBM-I training is undertaken, ensuring personal relevance of materials is likely to increase engagement and prevent attrition. In addition to potentially reducing RNT and psychological distress, given evidence that RNT predicts postnatal depression (e.g., Egan et al., 2017; O’Mahen et al., 2010) and predicts increases in depression from the last trimester of pregnancy to 28 weeks postpartum (Barnum et al., 2013), a further possibility that awaits testing in future research is that reducing antenatal RNT may prove effective in reducing the likelihood of suffering from postnatal depression and anxiety.

The study has some limitations. First, we cannot rule out the possibility that some participants in the non-pregnant group were trying to conceive, had recently miscarried, or were unknowingly pregnant at the time of participation. Whilst possible, given our large sample, we reason that the number of such participants is likely to be a very small proportion of the sample, and as such, do not expect that they would influence our findings. Second, framing the pregnancy-related scenarios in the first-person (common practice in the interpretation literature) may have inadvertently resulted in them being processed as personally relevant/salient by non-pregnant participants, despite the lack of relevance of the content (i.e., pregnancy) to their real day-to-day lives. Future studies which include self-relevant, non-self-relevant (presented in the first person) and non-self-relevant (referring to other) scenarios are needed to clarify this issue (see Wisco & Nolen-Hoeksema, 2010, for this distinction). Third, although our pregnant and non-pregnant samples were matched on levels of trait RNT and worry, groups differed on levels of self-reported rumination. Furthermore, mean levels of worry were higher than those reported in the general population, with a community sample of adults scoring 42.67 on the PSWQ (Startup & Erickson, 2006), compared to 52.78 in the current sample. Thus, we acknowledge that our sample may not be representative of the general population. Critically, however, this difference does not prevent us from answering our key research question. Fourth, whilst we checked that non-pregnant participants were not currently trying to fall pregnant, it is possible that for some of them, pregnancy may have in fact been personally relevant (e.g., if a close family member was pregnant). However, if this were the case it is likely that it only applied to a sub-group of the non-pregnant sample, and as such is unlikely to account for the current findings.

In any case, such limitations are balanced by notable strengths; for example, we conducted PPI with pregnant women to ensure that our pregnancy-related materials were relevant to the concerns of pregnant women, and thus maximise the ecological validity of the results. Furthermore, our findings are broadly replicated across two measures of interpretation bias, and demonstrate associations with different forms of repetitive negative thinking, as well as anxiety and depressed mood in pregnant women.

An interesting direction for future research in this area would be to investigate the possibility that pregnancy – a period characterised by uncertainty and ambiguous information for many women - exacerbates interpretation biases which were present prior to falling pregnant. For example, prospectively examining a sample of women of child-bearing age and re-assessing them during pregnancy would establish whether pre-existing biases are amplified during pregnancy, as well as shed light on the extent to which interpretation biases potentially interact with other cognitive processes (e.g., the tendency to attend to threat), as well as with life events more broadly.

In sum, this study is the first to investigate the interrelationship of negative interpretation bias, RNT, depression and anxiety in the perinatal period, and found positive associations between all of these variables. For pregnant women, interpretation bias was evident to the same degree for both material that was likely to be salient (pregnancy-related) and material that was general, and did not differ from that of non-pregnant women. Our finding that trait RNT is associated with interpretation bias for all ambiguous material, and across the full sample, underscores the need for novel interventions to target negative interpretations and reduce RNT in those at risk of developing clinical disorders characterised by unhelpful RNT. Given the wider impact of perinatal mental health problems on children, partners and the unborn child, we consider pregnant women a priority for RNT-focused preventive interventions.

## Supplementary Materials

The Supplementary Materials contain the following items (for access see Index of Supplementary Materials below):

- Appendix A: Example of materials in Scrambled Sentences Test
- Appendix B: Example of a pregnancy specific materials in the Recognition Test

10.23668/psycharchives.4428 Supplement 1 Supplementary materials to "Repetitive negative thinking and interpretation bias in pregnancy"

HirschC. R.
MeetenF.
GordonC.
NewbyJ. M.
BickD.
MouldsM. L.
 (2020). Supplementary materials to "Repetitive negative thinking and interpretation bias in pregnancy"
[Appendices]. PsychOpen. 10.23668/psycharchives.4428PMC964546636398060
