# Supplementary material for: Repetitive Negative Thinking and Interpretation Bias in Pregnancy
Source: Clin Psychol Eur. 2020 Dec 23;2(4):e3615. doi: 10.32872/cpe.v2i4.3615 (PMC9645466; doi:10.32872/cpe.v2i4.3615)
Supplement: Supplement 1 [file cpe-02-3615-s1.pdf]

Repetitive Negative Thinking and Interpretation Bias in Pregnancy

Hirsch, C.R., Meeten, F., Gordon, C., Newby, J.M., Bick, D., & Moulds, M.L.

Appendix B

Recognition Test

An example pregnancy related scenario is:

Stomach cramps

You are pregnant and over the last 2 days you have been feeling some stomach cramping, so you make an appointment to meet with your GP. You describe the cramps to the GP, and they explain whether or not they think it is a problem.

Question: Have you been feeling pain in your chest? (Correct answer: No)

For the above scenario, in the second section of the task, participants were presented with the following statements which they rated on a on a 4-item Likert scale from 1 (*very different in meaning*) to 4 (*very similar in meaning*).:

- Your GP says it is normal during pregnancy and you shouldn't be concerned (positive target)
- Your GP is concerned and wants further investigations (negative target)
- You have a much shorter wait to see the GP than you were expecting (positive foil)
- Your GP seems rushed and you don't feel they are listening (negative foil)
